# Pulmonary Embolism Response Team utilization during the COVID-19 pandemic

**DOI:** 10.1177/1358863X21995896

**Published:** 2021-04-04

**Authors:** Matthew T Finn, Shawn Gogia, Joseph J Ingrassia, Matthew Cohen, Mahesh V Madhavan, Shayan Nabavi Nouri, Yevgeniy Brailovsky, Amir Masoumi, Justin A Fried, Nir Uriel, Cara I Agerstrand, Andrew Eisenberger, Andrew J Einstein, Daniel Brodie, Erika B Rosenzweig, Martin B Leon, Koji Takeda, Anthony Pucillo, Philip Green, Ajay J Kirtane, Sahil A Parikh, Sanjum S Sethi

**Affiliations:** 1 Department of Cardiology, Columbia Irving Medical Center, New York, NY, USA; 2 Cardiovascular Research Foundation, New York, NY, USA; 3 Inteventional Cardiology, Hartford Hospital, Hartford, CT, USA; 4 Department of Pulmonology, Columbia Irving Medical Center, New York, NY, USA; 5 Department of Hematology/Oncology, Columbia Irving Medical Center, New York, NY, USA; 6 Department of Pediatric Cardiology, Columbia Irving Medical Center, New York, NY, USA; 7 Department of Cardiothoracic Surgery, Columbia Irving Medical Center, New York, NY, USA

**Keywords:** COVID-19, pulmonary embolism response team (PERT), venous thromboembolism, SARS-CoV-2

## Abstract

Coronavirus disease 2019 (COVID-19) may predispose patients to venous thromboembolism (VTE). Limited data are available on the utilization of the Pulmonary Embolism Response Team (PERT) in the setting of the COVID-19 global pandemic. We performed a single-center study to evaluate treatment, mortality, and bleeding outcomes in patients who received PERT consultations in March and April 2020, compared to historical controls from the same period in 2019. Clinical data were abstracted from the electronic medical record. The primary study endpoints were inpatient mortality and GUSTO moderate-to-severe bleeding. The frequency of PERT utilization was nearly threefold higher during March and April 2020 (*n* = 74) compared to the same period in 2019 (*n* = 26). During the COVID-19 pandemic, there was significantly less PERT-guided invasive treatment (5.5% vs 23.1%, *p* = 0.02) with a numerical but not statistically significant trend toward an increase in the use of systemic fibrinolytic therapy (13.5% vs 3.9%, *p* = 0.3). There were nonsignificant trends toward higher in-hospital mortality or moderate-to-severe bleeding in patients receiving PERT consultations during the COVID-19 period compared to historical controls (mortality 14.9% vs 3.9%, *p* = 0.18 and moderate-to-severe bleeding 35.1% vs 19.2%, *p* = 0.13). In conclusion, PERT utilization was nearly threefold higher during the COVID-19 pandemic than during the historical control period. Among patients evaluated by PERT, in-hospital mortality or moderate-to-severe bleeding were not significantly different, despite being numerically higher, while invasive therapy was utilized less frequently during the COVID-19 pandemic.

## Introduction

During the coronavirus disease 2019 (COVID-19) pandemic, prolonged immobilization and hypercoagulability have resulted in high rates of reported venous thromboembolism (VTE).^1–7^ A recent study from three centers in the Netherlands examining 184 patients in the intensive care unit showed a VTE prevalence of 27%.^8^ A second study demonstrated a 20.6% rate of VTE in COVID-19 patients compared to a 6.1% rate in historical controls.^9^

The Pulmonary Embolism Response Team (PERT) has become an integral part of pulmonary embolism and deep venous thrombosis care provided by many hospitals.^10–12^ The effect of COVID-19 on PERT response has not been described. We assessed our PERT activations, diagnoses, and treatments at New York-Presbyterian Hospital/Columbia University Irving Medical Center during the peak of the COVID-19 pandemic and compared them to historical controls.

## Methods

### Study design, population, and data sources

The study examined PERT consultation requests from March 1, 2020 through April 30, 2020 during the peak admission period of the COVID-19 pandemic at our hospital. Historical controls were taken from the same time period in the preceding year (2019). PERT members at our center are of an interdisciplinary team of interventional cardiology, vascular medicine, pulmonary and critical care, hematology, pulmonary hypertension, pharmacy, and cardiothoracic surgery. Consults are placed in the electronic medical record system triggering an immediate clinical evaluation and subsequent multidisciplinary discussion via a PERT telephone meeting, as required. Patients were seen and evaluated in the hospital by a member of the PERT consultative service. Physical examination and testing were limited during the 2020 period to limit staff exposure. Patient data from consultations during the index period were abstracted by chart review. Bleeding outcomes were collected and recorded according to the Global Utilization of Streptokinase and Tissue Plasminogen Activator for Occluded Coronary Arteries (GUSTO) scale, as well as the Bleeding Academic Research Consortium (BARC) scale.^13–15^ Data collection was closed for analysis on May 3, 2020 and subjects who were still inpatients were recorded as such in the database. May 2020 was chosen as the end of the analysis, as cases were rapidly declining during this period in New York and the study aimed to analyze data from the initial wave of the pandemic at our center.

The primary efficacy endpoint of the study was in-hospital mortality and the primary safety endpoint was GUSTO moderate or severe bleeding. Secondary endpoints were the rate of COVID-19 infection, anticoagulant use, and treatment strategy. Invasive treatments were defined as catheter-based strategies performed in the cardiac catheterization lab, operating room, or interventional radiology suite. These include mechanical thrombectomy (catheter or surgical) and catheter-directed lysis. Submassive VTE was defined as hemodynamically stable patients with signs of right ventricular injury as manifested by any of the following: troponin elevation greater than the laboratory cut off, NT-proBNP > 50 pg/mL, at least moderate right ventricle (RV) dilation/dysfunction or positive McConnell’s sign documented on echocardiogram reports/clinical documentation. Massive VTE was defined as any of the following: sustained systolic blood pressure (BP) < 90 mmHg, or use of vasopressors to maintain systolic BP > 90 mmHg, frank cardiogenic shock, cardiac arrest from VTE.^16^ Patients with a PERT consultation without imaging evidence of VTE were not included in the submassive/massive pulmonary embolism categories.

The study was approved by the institutional review board at Columbia University Irving Medical Center; due to the retrospective nature of the analysis, a waiver of patient consent was granted. The study was investigator initiated and was performed without outside funding. The investigators had direct access to the primary data and performed all analyses independently.

### Statistical analysis

Normally distributed continuous variables were reported as means with SD and compared with the Student’s *t*-test. Continuous variables that were non-normally distributed were reported as median with first and third quartiles and compared using the Mann–Whitney *U*-test. Categorical variables were summarized as percentages and were compared using chi-squared or Fisher’s exact test, as appropriate. Results were reported as odds ratios (OR) with 95% CI. Two multivariable logistic regression models were created to examine independent predictors of the primary outcome. Candidate variables were parsimoniously selected based on prior literature and included COVID-19 positive status, age, sex, obesity, creatinine, diabetes mellitus, deep venous thrombosis, chronic lung disease, cardiac biomarker positivity, transthoracic echocardiographic right ventricular size, and massive/submassive PE. Of these, included variables were those with a *p*-value less than 0.20 in addition to age, sex, creatinine, and diabetic status. Statistical analysis was performed with SAS software, version 9.4 (SAS Institute Inc., Cary, NC, USA).

## Results

### Study population and baseline characteristics

**Figure 1.**
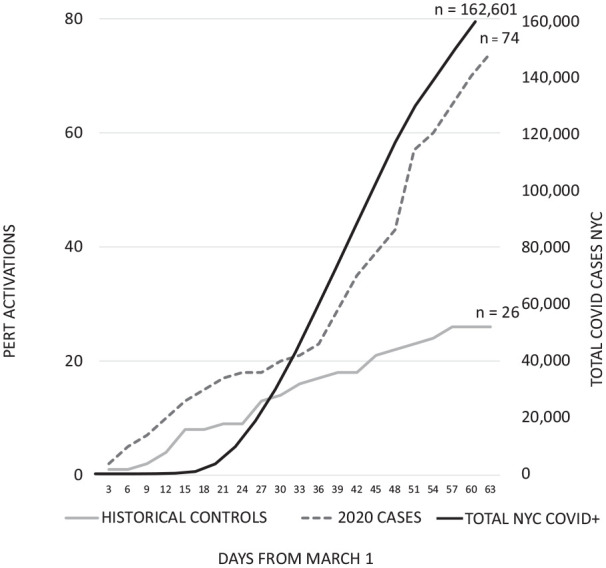
PERT consult volume March – April 2019 versus 2020 compared to New York City COVID-19 diagnoses. Note: Total New York COVID-19 positive cases represent a 7-day rolling average. Data obtained from NYC.gov (last access May 17, 2020). NYC, New York City; PERT, pulmonary embolism response team.

One hundred total consults were included in the study, encompassing PERT consultations from two periods: March – April 2019 (*n* = 26) and March – April 2020 (*n* = 74). In 2020, PERT consults were 2.8 times more common than in 2019 (*n* = 26; Figure 1 and Table 1). The slope of the PERT consults per 3-day period mirrored the overall rate of COVID injection in New York from Day 30 until the end of the study (log transformed slope of PERT consults: *m* = 0.16 vs *m* = 0.13). Patients undergoing PERT consultation in 2020 were more likely male and with a significantly lower rate of baseline chronic thromboembolic pulmonary hypertension (15.4%, *n* = 4 vs 1.4%, *n* = 1; *p* = 0.02). There was no difference in obesity status between 2020 cases and historical controls.

**Table 1.** Baseline characteristics: 2019 historical controls versus 2020 COVID-19 era (March-April 2020).

	2019 Historical controls	2020 COVID-19 era	*p*-value
	*n* = 26	*n* = 74	
Age, years	61.0 ± 20.1	58.6 ± 14.9	0.55
Male	38.5 (10)	60.8 (45)	0.05
BMI	33.8 ± 14.5	30.83 ± 6.4	0.31
Obese	61.5 (16)	44.6 (33)	0.14
Morbid obesity	23.1 (6)	20.3 (15)	0.76
Hypertension	69.2 (18)	52.7 (39)	0.14
Diabetes mellitus	26.9 (7)	35.1 (26)	0.44
Chronic lung disease	26.9 (7)	16.2 (12)	0.42
CTEPH	15.4 (4)	1.4 (1)	0.02
Congestive heart failure	7.7 (2)	1.4 (1)	0.16
Current or former tobacco	19.2 (5)	23.0 (17)	0.69
CVA	3.9 (1)	5.4 (4)	1
Creatinine ⩾ 2 mg/dL	11.5 (3)	18.9 (14)	0.4
ESRD	0 (0)	4 (5.4)	0.57

Data are presented as mean ± SD or % (*n*).

BMI, body mass index; COVID-19, coronavirus disease 2019; CTEPH, chronic thromboembolic pulmonary hypertension; CVA, cerebral vascular accident; ESRD, end stage renal disease.

**Figure 2.**
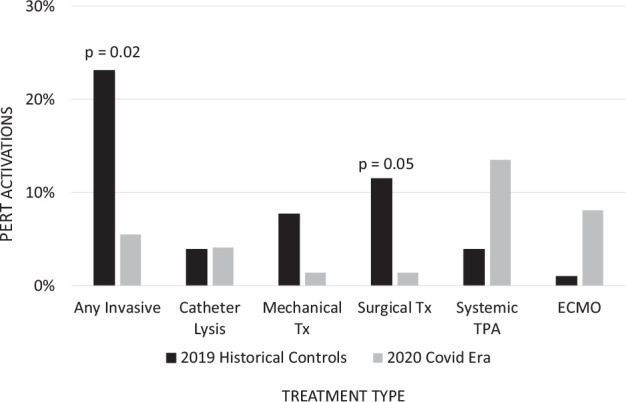
PERT venous thromboembolism treatments. COVID era: March-April 2020. COVID, coronavirus disease; ECMO, extracorporeal membrane oxygenation; PERT, pulmonary embolism response team; TPA, tissue plasminogen activator; Tx, thrombectomy.

In 2020, 60.8% of overall PERT activations were testing positive for COVID-19 by nasal swab polymerase chain reaction. After April 1, 2020, this percentage rose to 72.9%. During March and April, 2020 the definitive diagnosis of pulmonary embolism with computed tomography (CT) imaging was significantly less than for historical controls (58.1% vs 92.3%, *p* = 0.001; Table 2 and Figure 2). None of the 2020 PERT consult patients were diagnosed by ventilation-perfusion scintigraphy. Echocardiographic imaging identified five patients with mobile RV thrombus versus one in the historical controls. There was no difference in moderate-to-severe right ventricular enlargement on echocardiogram between groups; however, there was a higher rate of McConnell’s sign reported in the 2020 cases (56.5% vs 40.0%, *p* = 0.05).

**Table 2.** Presentation characteristics.

	2019 Historical controls	2020 COVID-19 era^a^	*p*-value
	*n* = 26	*n* = 74	
COVID-19 confirmed	0 (0)	60.8 (45)	<0.0001
Temp ⩾ 38°C	3.9 (1)	13.5 (10)	0.3
Heart rate peak (bpm)^b^	105.0 [92.0, 119.0]	110.5 [100.0, 124.0]	0.25
SBP low (mmHg)^b^	108.5 [86.0, 118.0]	104.5 [93.0, 117.0]	0.77
Supplemental O_2_%	53.9 (14)	74.3 (55)	0.05
Positive cardiac biomarkers^c^	67.6 (50)	69.2 (18)	0.89
D-dimer (μg/mL)^b^	16.2 [4.9, 17.2]	13.4 [4.4, 20.0]	0.74
ESR (mm/h)^b^	85.0 [71.0, 93.0]	92.0 [59.0, 121.0]	0.42
CRP (mg/dL)^b^	65.6 [6.4, 95.2]	227.0 [70.1, 300.0]	0.07
CT diagnosis	92.3 (24)	58.1 (43)	0.001
VQ diagnosis	3.9 (1)	0 (0)	0.26
Any DVT	50.0 (13)	33.8 (25)	0.14
Proximal DVT	46.2 (12)	31.1 (23)	0.17
sPESI	65.4 (17)	69.1 (38)	0.22
PESI	114.1 ± 50.2	118.8 ± 41	0.64
Submassive or massive	64.0 (16/25)	67.4 (33/49)	0.77
Recent trauma/surgery	19.2 (5)	10.8 (8)	0.27
Recent immobilization	30.8 (8)	20.3 (15)	0.27
Hormone use	0 (0)	1.35 (1)	1
Malignancy current or prior	7.7 (2)	12.2 (9)	0.72
Prior VTE	23.1 (6)	14.9 (11)	0.33
Echocardiographic data			
TTE RV size ⩾ mod^d^	52.0 (13)	43.1 (28)	0.49
McConnell’s sign	40.0 (10/25)	56.5 (13/65)	0.05
PASP (mmHg)	43.8 ± 14.5	38.8 ± 13.5	0.54
Right heart thrombus	4.0 (1/25)	7.7 (5/65)	1
CT data			
RV strain on CT	65.2 (15/23)	65.1 (28/43)	0.99
RV:LV^b^	1.19 [1.02, 1.42]	1.27 [1.06, 1.42]	0.38
PE location			
Saddle/main PA	17.4 (4/23)	20.9 (9/43)	1
Right, left or bilateral main PA	56.5 (13)	32.6 (14/43)	0.06
Lobar/seg arteries	26.1 (6)	39.5 (17)	0.27

Data are presented as mean ± SD or % (*n*) unless otherwise noted.

a March-April 2020

b Median [IQR].

c Tn T > 0.04 ng/mL; high-sensitivity Tn > 14 ng/dL ng/L; NT-ProBNP: age less than 50 years < 450 pg/mL; age 50–70 < 180 pg/mL.

d Based on visually estimated ejection fraction as read in clinically reported echocardiograms.

bpm, beats per minute; COVID-19, coronavirus disease 2019; CRP, C-reactive protein; CT, computed tomography; DVT, deep venous thrombosis; ESR, erythrocyte sedimentation rate; LV, left ventricle; O_2_, oxygen; PA, pulmonary artery; PASP, pulmonary artery systolic pressure; PESI, pulmonary embolism severity index; RV, right ventricle; SBP, systolic blood pressure; Seg, segmental; sPESI, simplified pulmonary embolism severity index; TTE, transthoracic echocardiogram; VQ, ventilation perfusion; VTE, venous thromboembolism.

### Treatments and outcomes

**Figure 3.**
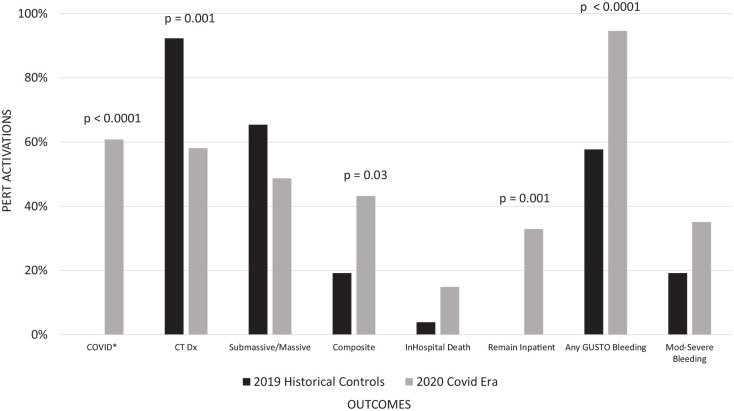
Major outcomes with PERT consultation. COVID era: March-April 2020. Note: ‘remain inpatient’ represents the percentage of patients who were still hospitalized as of the closure of the dataset on May 3, 2020. COVID, coronavirus disease; CT, computed tomography; Dx, diagnosis; GUSTO, Global Utilization of Streptokinase and Tissue Plasminogen Activator for Occluded Coronary Arteries; Mod, moderate; PERT, pulmonary embolism response team.

There was no difference in the rate of intensive care unit admission between the groups. However, COVID-19 era patients were significantly more likely to require mechanical ventilation (47.3% vs 15.4%, *p* = 0.04) or a vasoactive medication (50.0% vs 19.2%, *p* = 0.006). The use of extracorporeal membrane oxygenation (ECMO) was 8.1% in the COVID-19 era patients versus 0% in the historical control group (*p* = 0.33; Table 3 and Figure 3). Of these, four patients had veno-venous, two patients had veno-arterial, and one patient had both. Four of six patients on ECMO survived to discharge.

**Table 3.** Treatment and outcomes.

	2019 Historical controls	2020 COVID-19 era	OR (95% CI)	*p*-value
	*n* =26	*n* = 74		
Intensive care				
ICU	61.5 (16)	62.2 (46)	1.93 (0.41–2.57)	0.96
Mechanical ventilation	15.4 (4)	47.3 (35)	4.9 (1.54–15.73	0.04
Vasoactive medications	19.2 (5)	50.0 (37)	4.2 (1.43–12.32)	0.006
ECMO	0 (0)	8.1 (6)	5.02 (0.27–92.4)	0.33
Treatment strategy				
Any invasive	23.1 (6)	5.5 (4)	0.19 (0.05–0.74)	0.02
Surgical thrombectomy	11.5 (3)	1.4 (1)	0.11 (0.01–1.10)	0.05
Catheter thrombectomy	7.7 (2)	1.4 (1)	0.16 (0.10–1.89)	0.16
Catheter-directed lysis	3.9 (1)	4.1 (3)	1.06 (0.11–10.63)	1
Systemic lysis	3.9 (1)	13.5 (10)	3.9 (0.48–32.12)	0.3
Anticoagulation alone	46.5 (12)	83.3 (60)	5.0 (1.90–13.14)	0.0006
Major endpoints				
Composite	19.2 (5)	43.2 (32)	3.20 (1.09–9.41)	0.03
In-hospital mortality	3.9 (1)	14.9 (11)	4.37 (0.53–35.60)	0.18
Remain inpatient	0.0 (0)	32.9 (24)	26.2 (1.53–448.72)	0.0008
Length of stay^a^	4.0 [3.2, 16.4]	5.7 [2.9, 12.8]	0.99 (0.94–1.04)	0.92
Initial inpatient anticoagulation				
Heparin	80.8 (21)	71.6 (53)	0.60 (0.20–1.8)	0.36
Enoxaparin	11.5 (3)	21.6 (16)	2.11 (0.60–8.0)	0.40
Other	7.7 (2)	6.8 (5)	0.86 (0.16–4.78)	1
Discharge anticoagulation				
NOAC	60.9 (14/23)	81.8 (27/33)	2.89 (0.86–9.78)	0.08
Enoxaparin	13.0 (3/23)	15.2 (5/33)	1.19 (0.25–5.56)	1
Warfarin	26.1 (6/23)	3.0 (1/33)	0.09 (0.01–0.8)	0.02
GUSTO bleeding				
GUSTO severe	0 (0)	5.4 (4)	3.38 (0.18–65.00)	0.57
GUSTO moderate–severe	19.2 (5)	35.1 (26)	2.27 (0.77–6.74)	0.13
GUSTO moderate	19.2 (5)	29.7 (22)	1.78 (0.59–5.31)	0.3
GUSTO mild	38.5 (10)	59.5 (44)	2.34 (0.94–5.87)	0.07
GUSTO any	57.7 (15)	94.6 (70)	12.8 (3.59–45.84)	<0.001
BARC bleeding				
BARC major^b^	0 (0)	4.1 (3)	2.59 (0.12–51.9)	0.57
BARC minor	57.7 (15)	91.9 (68)	8.31 (2.65–26.01)	<0.0001
BARC I	26.9 (7)	24.32 (18)	0.87 (0.31–2.41)	0.79
BARC II	11.5 (3)	35.1 (26)	4.2 (1.13–15.15)	0.02
BARC IIIa	19.2 (5)	32.4 (74)	2.02 (0.68–6.00)	0.2
BARC IIIIb	0 (0)	1.4 (1)	1.08 (0.04–27.4)	0.55
BARC V	0 (0)	2.7 (2)	1.83 (0.08–39.3)	1

Data are presented as % (*n*) unless otherwise noted.

a Median [IQR].

b No BARC IV events occurred.

BARC, Bleeding Academic Research Consortium; COVID-19, coronavirus disease 2019; ECMO, extracorporeal membrane oxygenation; GUSTO, Global Utilization of Streptokinase and Tissue Plasminogen Activator for Occluded Coronary Arteries; ICU, intensive care unit; NOAC, novel oral anticoagulation.

### Major endpoints

Invasive treatment strategies were used less often during the COVID-19 era (5.5% vs 23.1%, *p* = 0.02), with all invasive interventions occurring in COVID negative patients. Conversely, there was a nonsignificant increase in the use of systemic fibrinolytic therapy in patients with COVID-19 (13.5% vs 3.9%, *p* = 0.3; Table 3 and Figure 3).

There were higher frequencies of in-hospital mortality or moderate-to-severe bleeding in patients receiving PERT consultations during the COVID-19 period compared to historical controls (mortality 14.9% vs 3.9%, *p* = 0.18, moderate-to-severe bleeding 35.1% vs 19.2%, *p* = 0.13), but these differences did not meet statistical significance. BARC scale major bleeding was not different between groups; however, a higher rate of BARC minor bleeding was seen in the COVID-19 era patients (91.9% vs 57.7%, *p* < 0.0001). There were two BARC V fatal bleeding events and one intracranial hemorrhage – both occurring in the COVID-19 era group.

On multivariable logistic regression analysis, only COVID-19 positive status (OR 9.1, 95% CI: 1.44–57.51, *p* = 0.02), age (OR 0.89, 95% CI: 0.82–0.97, *p* = 0.008), and body mass index (OR 0.88, 95% CI: 0.80–0.97, *p* = 0.01) were associated with inpatient mortality (Table 4).

**Table 4.** Multivariable modeling: covariate relationship to inpatient mortality.

	Adjusted OR	95% CI	*p*-value
COVID-19 positive	9.1	1.44–57.51	0.02
Male	1.0	0.21–5.63	0.93
Age	0.89	0.82–0.97	0.008
BMI	0.88	0.80–0.97	0.01
Initial creatinine (mg/dL)	1.01	0.94–1.08	0.89
Diabetes mellitus	0.67	0.16–2.70	0.57
Chronic lung disease	2.1	0.22–20.82	0.52

BMI, body mass index; COVID-19, coronavirus disease 2019; OR, odds ratio.

### Systemic fibrinolytic utilization and right heart thrombus

The use of systemic fibrinolytic therapy (*n* = 11) was associated with a higher rate of in-hospital mortality (54.5%, 6/11, *p* < 0.001) in the overall study. Slightly over half of patients who were given systemic lytic therapy had confirmed pulmonary embolism or clot in transit (*n* = 6/11). Of the fibrinolysis patients, 27.3% (3/11) had GUSTO severe bleeding and 63.6% (7/11) had GUSTO moderate-to-severe bleeding. Among patients who received systemic thrombolysis and had a GUSTO moderate-to-severe bleeding event, 66.7% died, including one patient who suffered an intracranial hemorrhage.

There were six cases of right heart thrombus; five occurring in COVID-19 positive patients. Two of these patients received systemic fibrinolysis, one was placed on veno-venous ECMO, and the remaining were treated with anticoagulation alone. At the time of closure of the study for data analysis, three of these six patients had died and the three who remained alive were still hospitalized.

## Discussion

The COVID-19 pandemic created a surge of critically ill patients, particularly in New York City, where the number of individuals infected was the highest of major metropolitan areas in the US.^17^ Suspected increase in rates of VTE or suspected VTE are due to a variety of possible mechanisms, such as hypercoagulability, prolonged immobility, and inflammation of pulmonary vasculature leading to immunothrombosis.^3^ Our study sought to evaluate PERT consultation, treatment, and in-hospital outcomes during the COVID-19 era. The main conclusions from the study were: first, the rate of PERT consultation was almost three times higher compared to the prior year, despite a lower rate of confirmatory testing. Second, the rate of invasive therapies (surgical or catheter embolectomy or catheter-directed lysis) were lower during the COVID-19 era, corresponding with a rise in the frequency of systemic fibrinolytic therapy. Third, during the pandemic, there was a numerically but not statistically higher rate of GUSTO moderate-to-severe bleeding complications. There were no differences in the rates of GUSTO or BARC scale major bleeding events. Lastly, on multivariable analysis, COVID-19 infection, age, and body mass index (BMI) were associated with inpatient mortality, while traditional pulmonary embolism risk factors, such as severity of PE, categorical biomarker elevation, and degree of RV enlargement, did not reach statistical significance.

PERT consultation was an important aspect of comprehensive care provided during the COVID-19 era. During the month of March 2020, the rate of consultation initially was similar to the year prior, with a steep increase in consultations occurring at the beginning of April and continuing throughout the month. Consultations appeared to rise largely in parallel to the overall COVID-19 positive case volume in the city, with similar transformed slopes from day 30 onward.

PERT consultations during the COVID-19 pandemic occurred despite a significantly lower than normal definitive diagnosis of VTE. The reasons for reduced utilization of CT imaging likely include greater rates of COVID-19-related acute renal failure precluding contrast administration, severe systemic illness, and concern of staff exposure to the virus.^18–22^ For similar reasons, invasive treatments performed by the PERT occurred less often during COVID-19 and were not performed on COVID positive patients in the study. In response, there was a rise in the use of fibrinolytic therapy.

There were overall high rates of bleeding, particularly during the COVID-19 era. While these differences did not reach statistical significance (aside from BARC minor bleeding), it is important to note that over 30% of COVID-19 era patients remained inpatients at the end of the study, most of whom were on systemic anticoagulation. Therefore, further bleeding events are likely to occur with longer follow-up. Given both high rates of VTE and high rates of hemorrhagic complications, optimal prophylactic and treatment dosing are currently under investigation (clinicaltrials.gov NCT04367831). Furthermore, if recurrent episodes with COVID-19 or other emerging disease states occur, the role for invasive treatments must be reconsidered as an option to potentially reduce bleeding and improve overall outcomes.

On multivariable modeling, COVID-19 infection, age, and BMI were important factors associated with inpatient death. Traditional PE risk factors like severity of PE (massive/submassive) or degree of RV dysfunction were not significant in the adjusted model. This may be explained, first, by the likelihood of COVID-19 leading to death due to causes other than PE (such as severe acute respiratory distress syndrome); and second, by the small size of the analysis cohort reducing the power to determine adjusted effects.

### Study limitations

There were inherent limitations to this study. First, this is a single-center, retrospective dataset without a clinical endpoint committee for independent adjudication. The overall sample size of the analysis was limited and therefore underpowered for some analyses. Conclusions from these data should be viewed as primarily hypothesis generating. Second, unmeasured confounding may exist, as patients presenting during the COVID-19 pandemic had greater systemic illness and overall medical complexity with higher rates of mechanical ventilator and vasopressor use.^18,23^ Additionally, the rate of PERT consultation has steadily risen during the greater than 5-year existence of the service at our center; however, the increase seen in the study is far beyond the expected yearly increase, and is clearly associated with the pandemic in the New York area. Third, a large percentage of COVID-19 era patients were still inpatients at the time of closure of the database; therefore, endpoints in that group may be underestimated. Fourth, no control group in which patients with VTE who did not receive PERT consultation was available to assess the independent effect of the PERT team on outcomes during COVID-19. Last, pulmonary embolism was categorized according to massive and submassive criteria, rather than the now commonly utilized more granular definition dividing submassive PE into low, medium, and high-risk categories.

## Conclusions

PERT consultation increased almost threefold during the COVID-19 pandemic when compared to the historical control period. Despite this, PERT-guided invasive therapy was offered less frequently during the 2020 COVID-19 era. Among patients seen in PERT consultation, the individual rates of in-hospital mortality and GUSTO scale moderate-to-severe bleeding were numerically but not significantly higher during the COVID-19 pandemic than in historical controls. Further research is required to examine the impact of PERT consultations on outcomes compared to those without such consultations during the COVID-19 pandemic.
